# Construction and validation of a novel and superior protein risk model for prognosis prediction in esophageal cancer

**DOI:** 10.3389/fgene.2022.1055202

**Published:** 2022-11-15

**Authors:** Yang Liu, Miaomiao Wang, Yang Lu, Shuyan Zhang, Lin Kang, Guona Zheng, Yanan Ren, Xiaowan Guo, Huanfen Zhao, Han Hao

**Affiliations:** ^1^ Department of Pathology, Hebei General Hospital, Shijiazhuang, China; ^2^ Basic Medical College, Hebei Medical University, Shijiazhuang, China; ^3^ Department of Gynecology, Hebei General Hospital, Shijiazhuang, China; ^4^ Department of Radiology, Hebei General Hospital, Shijiazhuang, China; ^5^ Department of Pharmacology, The Key Laboratory of New Drug Pharmacology and Toxicology, Center of Innovative Drug Research and Evaluation, Hebei Medical University, Shijiazhuang, China

**Keywords:** esophageal cancer, proteomics, TCGA, prognosis, risk model, treatment

## Abstract

Esophageal cancer (EC) is recognized as one of the most common malignant tumors in the word. Based on the biological process of EC occurrence and development, exploring molecular biomarkers can provide a good guidance for predicting the risk, prognosis and treatment response of EC. Proteomics has been widely used as a technology that identifies, analyzes and quantitatively acquires the composition of all proteins in the target tissues. Proteomics characterization applied to construct a prognostic signature will help to explore effective biomarkers and discover new therapeutic targets for EC. This study showed that we established a 8 proteins risk model composed of ASNS, b-Catenin_pT41_S45, ARAF_pS299, SFRP1, Vinculin, MERIT40, BAK and Atg4B *via* multivariate Cox regression analysis of the proteome data in the Cancer Genome Atlas (TCGA) to predict the prognosis power of EC patients. The risk model had the best discrimination ability and could distinguish patients in the high- and low-risk groups by principal component analysis (PCA) analysis, and the high-risk patients had a poor survival status compared with the low-risk patients. It was confirmed as one independent and superior prognostic predictor by the receiver operating characteristic (ROC) curve and nomogram. K-M survival analysis was performed to investigate the relationship between the 8 proteins expressions and the overall survival. GSEA analysis showed KEGG and GO pathways enriched in the risk model, such as metabolic and cancer-related pathways. The high-risk group presented upregulation of dendritic cells resting, macrophages M2 and NK cells activated, downregulation of plasma cells, and multiple activated immune checkpoints. Most of the potential therapeutic drugs were more appropriate treatment for the low-risk patients. Through adequate analysis and verification, this 8 proteins risk model could act as a great prognostic evaluation for EC patients and provide new insight into the diagnosis and treatment of EC.

## Introduction

International Agency for Research on Cancer (IARC) global statistical report showed that in 2020, the number of new cases of esophageal cancer (EC) was 604,000, ranking 8th in the world. There were 544,000 new deaths from EC, ranking 6th in the death spectrum of malignant tumors worldwide. The incidence of EC showed obvious regional differences, which is mainly distributed in Asia and Africa, and higher in male population than that in female population (Sung et al., 2021). Most EC patients with unobvious early symptoms are diagnosed with advanced stage or distant metastases and have a poor prognosis. According to relevant studies, the 5-year survival rate of early patients can be reached 70% after effective treatment, and the 5-year survival rate of advanced patients is only 10%–30% (Kelly, 2019; Han et al., 2022). Therefore, the research of EC has great social value and profound practical significance.

Proteomic studies, an important part of the post-gene era, can analyze protein expression levels, post-translational modifications and protein-protein interactions in tissues and cells on a large scale. By comparing the proteome between normal and pathological individuals, some “disease-specific protein molecules” could be explored, which can provide molecular markers for early diagnosis or become molecular targets for new drug design of disease (Vogel and Marcotte, 2012; Suhre et al., 2021). In-depth understanding of the causes and mechanisms of tumors and finding tumor markers for early diagnosis have become the key to realize early diagnosis and treatment of tumors, and also been a hot spot in basic and clinical research of tumors. High-throughput proteomic analysis is used as the research technique to deeply explore the changes of protein expression during tumorigenesis and progression, which provides new ideas and new methods for the screening of tumor protein markers, the identification of clinical drug targets and the exploration of the molecular mechanism of tumors (Hanash et al., 2008; Tan et al., 2012). With the continuous accumulation of clinical information of EC, the data of EC is constantly improved. The effective use of EC medical data can help improve the diagnosis and therapy of EC patients, which plays an important role in promoting the development of intelligent medical treatment of EC (Triantafyllou and Wijnhoven, 2020). In this study, we aimed to construct a protein prediction risk model and evaluate its prognostic power for EC patients in this study, which could be helpful to discover new molecular biomarkers and select effective treatments for patients.

## Materials and methods

### Data collection

The proteome profiling containing 126 EC cases detected by Reverse Phase Protein Array (RPPA), and clinical data from 185 EC patients were obtained from the Cancer Genome Atlas (TCGA) database (https://portal.gdc.cancer.gov/repository) on 13 August 2022. After matching the proteome data and clinical data, we finally obtained 126 intersecting cases for further study.

### Screening prognosis-related proteins

Volcano plot of differentially expressed prognosis-related proteins (*p* < 0.05) between EC and normal tissues in the TCGA database was constructed *via* “dplyr”, “ggplot2”, “ggrepel” R packages. For univariate Cox regression analysis, forest plot was applied to screen prognosis-related proteins based on the survival state of EC patients (*p* < 0.05) *via* “survival”, “caret” and “glmnet” R packages. The least absolute shrinkage and selection operator (Lasso) regression analysis was used to further screen significant proteins by “survival”, “caret” and “glmnet” R packages (10-fold cross-validation, 1,000 cycles, *p* = 0.05).

### Construction and verification of the protein prognostic risk model

We randomized intersecting cases (group number = 1) into train and test groups with a 1:1 ratio. The protein prognostic risk model was constructed *via* multivariate Cox regression analysis in the train group *via* “survival”, “caret”, “glmnet”, “survminer” and “timeROC” R packages. The model formula was as follows: risk score = (Coeffcient Protein_1_ × Protein_1_ expression) + (Coeffcient Protein_2_ × Protein_2_ expression) +…+ (Coeffcient Protein_*n*_ × Protein_*n*_ expression), Where Coefcient represented the regression coefficient of multivariate Cox regression analysis for each protein. Based on the median risk score, all EC patients in the train and test groups were divided into the high- and low-risk groups. The overall survival, progression free survival, risk score and survival status of the high- and low-risk patients were analyzed *via* “survminer” and “survival” R packages. The heat maps of protein expressions were created by “pheatmap” R package. Principal component analysis (PCA) was used to distinguish the high-risk patients and low-risk patients by the “scatterplot3d” R package.

### Evaluation of the protein prognostic risk model

To evaluate the prognostic levels of EC patients grouped by the risk model and clinical features, univariate and multivariate Cox regression analyses and the receiver operating characteristic (ROC) curves in the train, test and entirety groups were performed by “survival”, “survminer” and “timeROC” R packages. The overall survival of high- and low-risk EC patients separated into different groups according to clinical characteristics were analyzed by “survival”, “survminer” R packages. The method of overall survival analysis according to protein expressions was the same as above.

### Nomogram and calibration

The data of age, gender, tumor grade, tumor stage, T stage, M stage, N stage and risk model were employed to construct a nomogram for the 1-, 2-, 3-year overall survival. According to the status of each EC patient, the point corresponding to each factor was added up, and the survival rates of 1-, 2- and 3- year could be predicted by the total points. Correction curves statistically analyzed by Hosmer-Lemeshow test were performed to verify the accuracy and consistency of the nomogram by “survival”, “regplot” and “rms” R packages.

### Gene set enrichment analyses analysis and protein interaction network

Through “limma”, “org.Hs.eg.db”, “cluster Profiler” and “enrichplot” R packages, the significantly enriched KEGG and GO pathways in the risk groups were explored by screening the gene set (c2. cp.kegg.symbols.gmt; c5. go.symbols) in gene set enrichment analyses (GSEA) 4.2.3 software (NOM *p value* < 0*.*05 and |NES| > 1.5). A circos plot was made to show the network of interactions among the proteins in the risk model by “corrplot” and “circlize” packages. Sankey diagram created by “survminer” and “survival” R packages visualized the mutually regulating relationship between the protein prognostic risk model and other proteins.

### Tumor immunology and exploration of potential therapeutic agents

To investigate the relationship between the risk model and tumor immune microenvironment, we calculated the infiltration values of EC dataset in TCGA through CIBERSORT (Chen et al., 2018). Immune cell infiltration status and immune checkpoints activation in the risk groups were analyzed by “limma”, “ggpubr”, “ggpubr” and “ggplot2” R packages, and radar plot was constructed by “fmsb” R package. According to Genomics of Drug Sensitivity in Cancer (GDSC, https://www.cancerrxgene.org/), the “pRRophetic”, “limma”, “ggpubr” and “ggplot2” R packages were applied to predict therapeutic compounds depending on half-maximal inhibitory concentration (IC50) of each EC patient.

## Results

**FIGURE 1:**
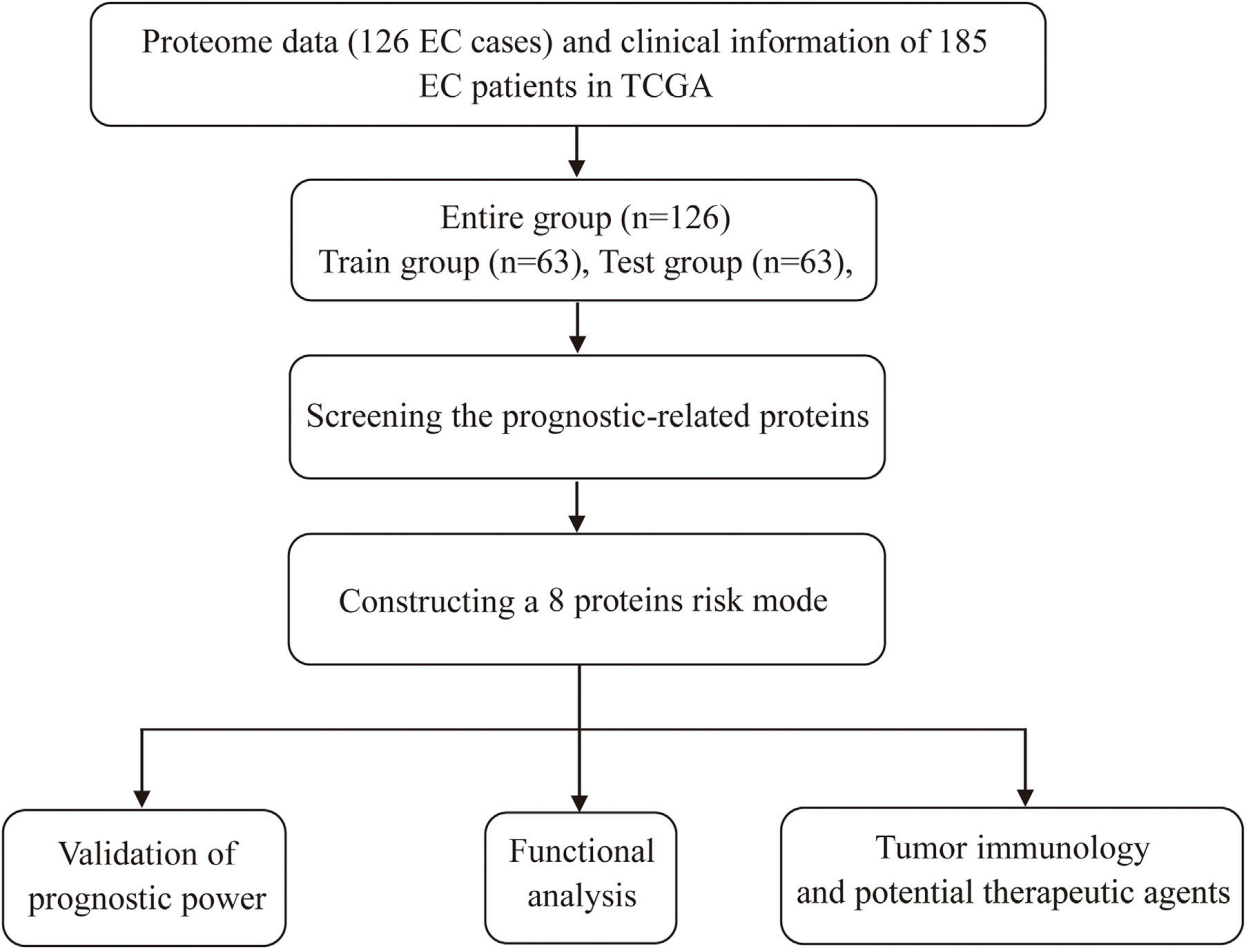
Schematic diagram of the research process.

**FIGURE 2:**
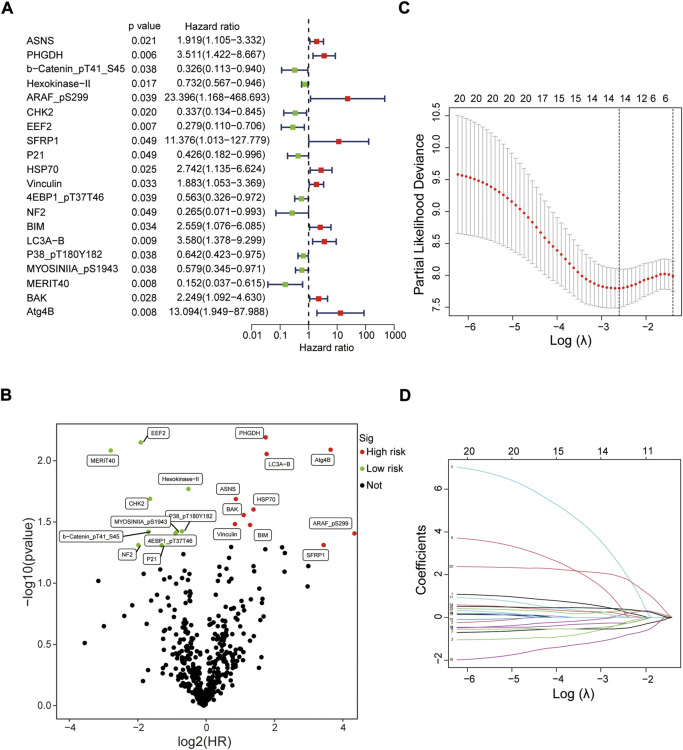
Screening prognostic-related proteins in EC from TCGA database. **(A)** Study of the correlation between 20 prognostic-related proteins and overall survival of EC patients by univariate Cox regression analysis. **(B)** The upregulated and downregulated prognostic-related proteins in volcano plot. **(C,D)** The Lasso regression analysis of these 20 proteins.

Screening prognostic-related proteins and constructing the risk model Schematic diagram of the research process was presented in Figure 1. The proteome profiling containing 126 EC cases (Supplementary Table S1) and clinical data from 185 EC patients (Supplementary Table S2) were received from TCGA database. After matching the two databases, we finally obtained 126 intersecting cases (Supplementary Table S3). Univariate Cox regression analysis showed 20 proteins (*p* < 0*.*05) significantly associated with survival time of EC patients (Figure 2A), all the significantly expressed proteins were displayed in a volcano plot, including 10 high risk proteins (Hazard ratio > 1) and 10 low risk proteins (Hazard ratio < 1, Figure 2B; Supplementary Table S4). 14 proteins related to survival time and survival state of EC patients were extracted through Lasso regression analysis on these 20 proteins (Figures 2C,D; Supplementary Table S5). To improve the accuracy of the protein prognostic risk model, we randomized 126 intersection cases (entire group, *n* = 126) into train group (*n* = 63) and test group (*n* = 63) with a 1:1 ratio. Subsequently, we constructed the prognostic risk model composed of 8 proteins (ASNS, b-Catenin_pT41_S45, ARAF_pS299, SFRP1, Vinculin, MERIT40, BAK and Atg4B) by multivariate Cox analysis in the train group (Table 1). The risk score = ASNS × (0.872) + b-Catenin_pT41_S45 × (-1.282) + ARAF_pS299 × (5.935) + SFRP1 × (2.947) + Vinculin × (0.809) + MERIT40 × (−2.095) + BAK × (0.748) + Atg4B × (2.625). The high-risk group and low-risk group were established depending on the median risk score.

**TABLE 1:** The protein prognostic risk model.

Protein	Coeffcient	HR	HR.95L	HR.95H	*p* value
ASNS	0.872	1.929	1.115	3.332	0.021
b-Catenin_pT41_S45	−1.282	0.326	0.113	0.940	0.038
ARAF_pS299	5.935	23.396	1.168	468.693	0.039
SFRP1	2.947	11.376	1.013	127.779	0.049
Vinculin	0.809	1.883	1.053	3.369	0.033
MERIT40	−2.095	0.152	0.037	0.615	0.008
BAK	0.748	2.249	1.092	4.630	0.028
Atg4B	2.625	13.093	1.949	87.987	0.008

HR, hazard ratio; L, low; H, high.

### Evaluation of the protein prognostic risk model based on the clinical characteristics

**FIGURE 3:**
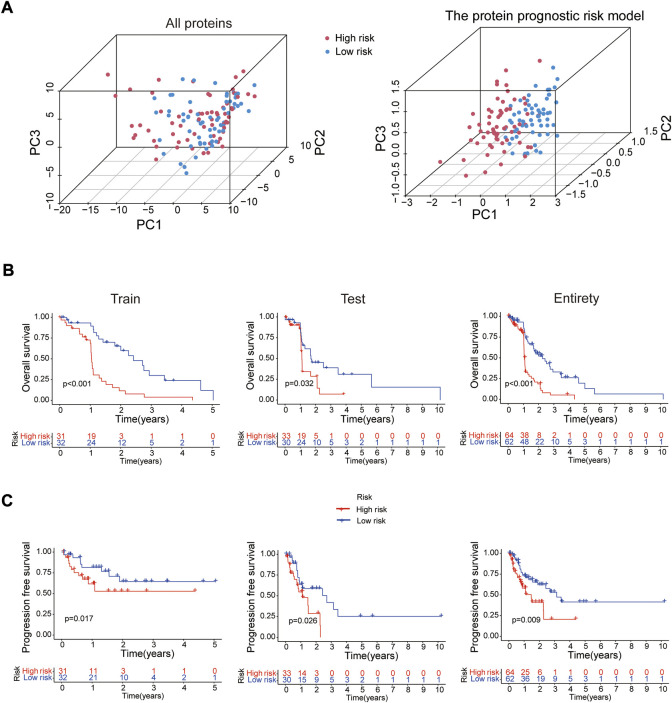
Survival analysis of the risk model. **(A)** PCA analysis for whole protein expression profile and the risk model. The overall survival **(B)** and progression free survival **(C)** analyses between the high- and low-risk groups in the train (*n* = 63), test (*n* = 63) and entire (*n* = 126) sets.

**FIGURE 4:**
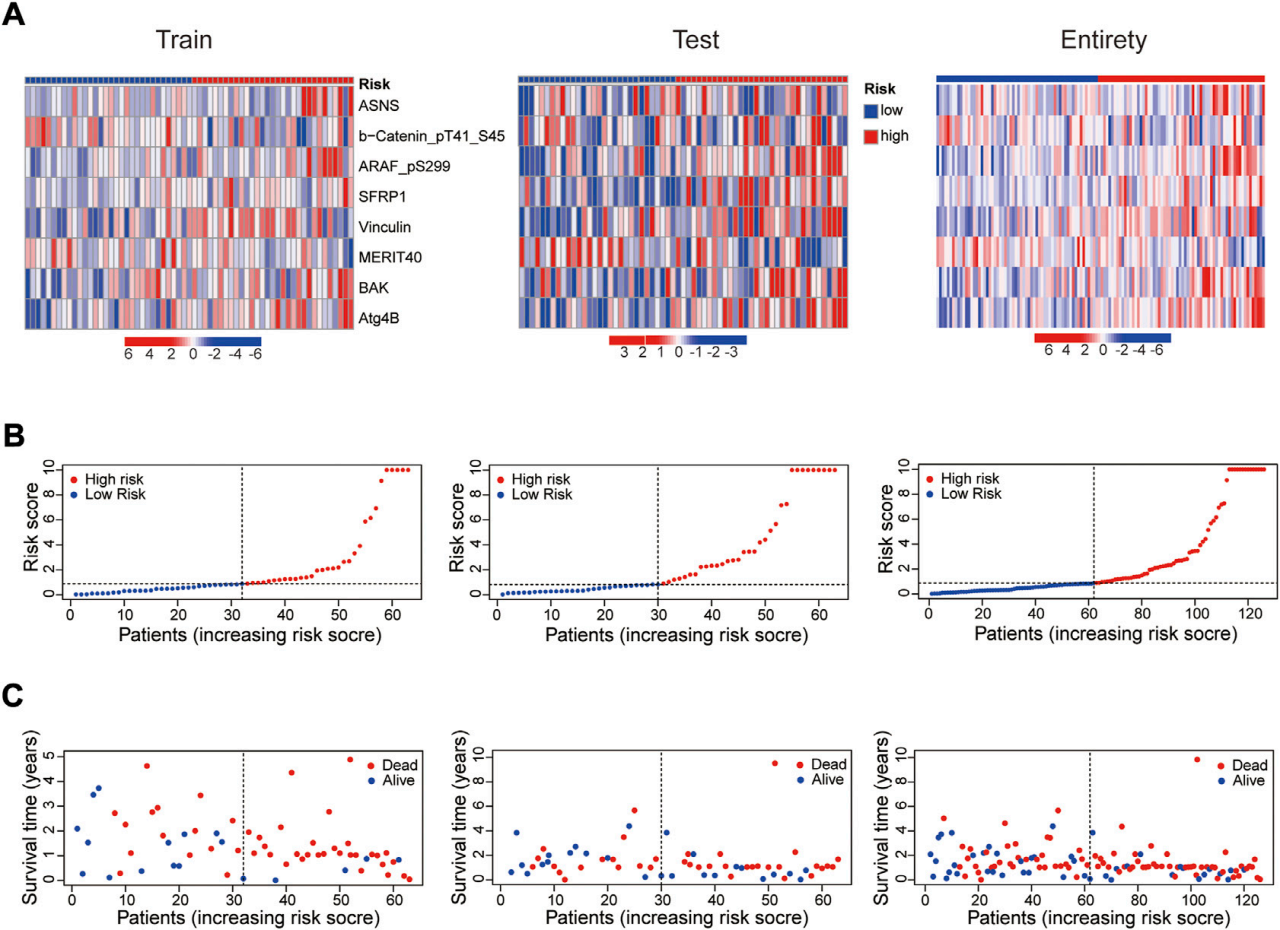
Prognosis evaluation of the established protein prognostic risk model. Heat maps of 8 prognostic-related proteins expressions **(A)**, the distribution of risk scores **(B)** and the survival time **(C)** between the high- and low-risk groups in the three sets.

In 2 expression profiles (whole protein expression profile and the protein prognostic risk model), PCA was applied to examine the differences between the high- and low-risk groups (Figure 3A). The result showed that the risk model had the best discrimination ability and could perfectly distinguish patients in the high- and low-risk groups. Next, the overall survival (Figure 3B) and progression free survival (Figure 3C) analyses in the train, test and entire groups indicated the high-risk EC patients had a worse prognosis than the low-risk EC patients. The relevant expressions of 8 proteins showed that ASNS, ARAF_pS299, SFRP1, Vinculin, BAK and Atg4B were highly expressed in the high-risk group, while b-Catenin_pT41_S45 and MERIT40 were highly expressed in the low-risk group (Figure 4A). The distribution of risk score and survival time of EC patients in the train, test and entire groups were compared between the high- and low-risk groups, all results demonstrated the patients with high risk had worse prognosis (Figures 4B,C).

**FIGURE 5:**
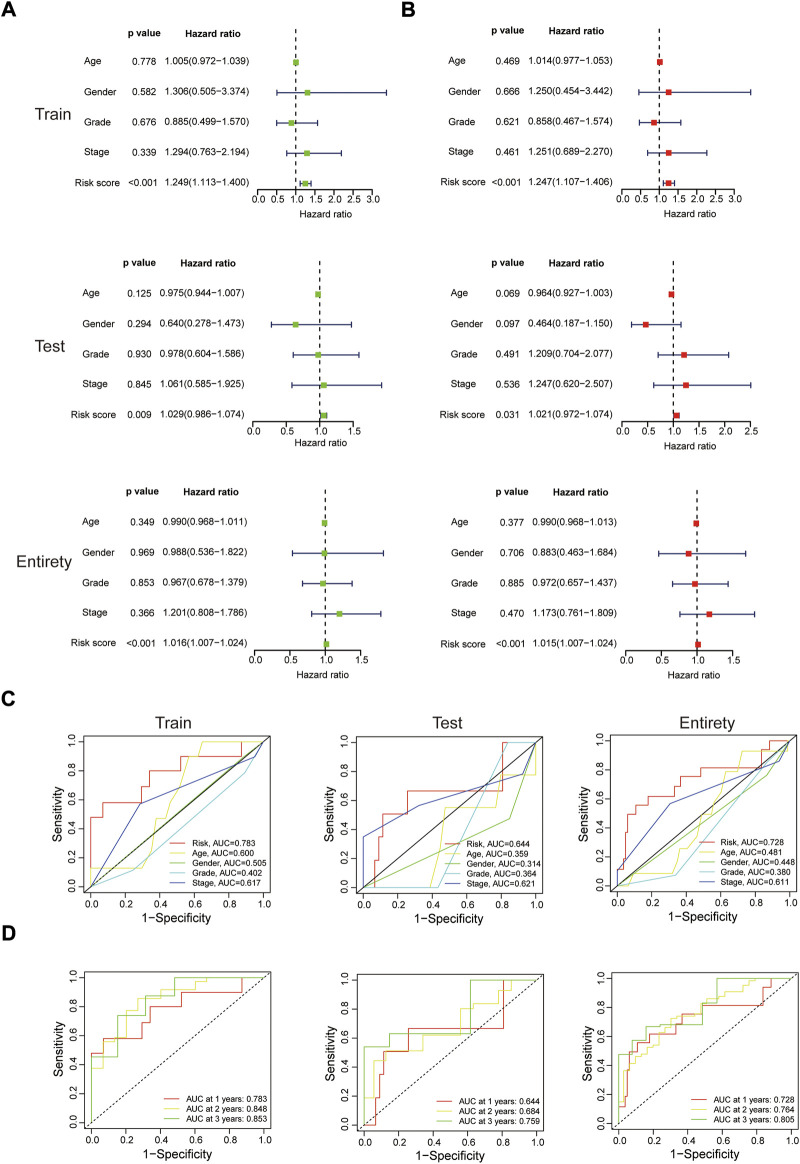
In-depth assessment of the protein prognostic risk model. **(A,B)** Univariate and multivariate Cox regression analyses of clinical features and the risk model with overall survival in the three sets. ROC analysis for 1 year survival rate of the risk model, age, gender, grade and stage **(C)**, and the risk model at 1-, 2-, 3-year survival time **(D)**.

**FIGURE 6:**
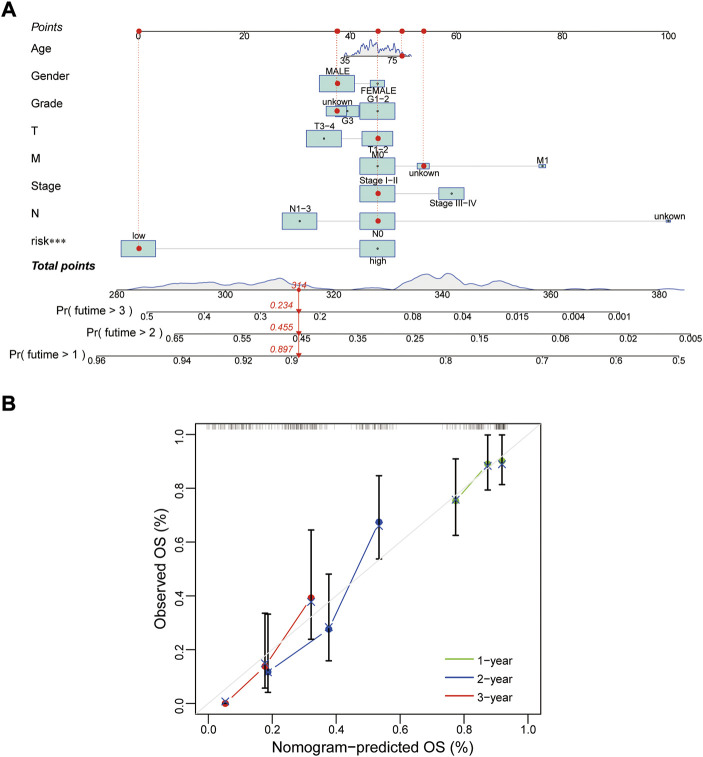
Construction of a nomogram. **(A,B)** Nomogram and calibration plot for predicting 1-, 2-, 3-year survival rate of EC patients. OS, overall survival.

Whether this protein risk model is an independent and efficient prognostic predictor for EC patients needs further in-depth verification. Compared with clinical features, univariate Cox analysis showed the hazard ratios of the risk score were 1.249, 1.029 and 1.016 respectively in the train, test and entire groups (all *p* < 0.05, Figure 5A), and 1.247, 1.021 and 1.015 respectively in multivariate Cox analysis (all *p* < 0.05, Figure 5B). The ROC curve, which of the outcome need to be explained by the area under the ROC curve (AUC), was used to assess survival rates of EC patients. At the 1-year ROC, the risk model in the train, test and entire groups were 0.783, 0.644 and 0.728, respectively, superior to other clinical factors (Figure 5C). The 1-, 2-, 3-year AUC were 0.783, 0.848 and 0.853 in the train group, 0.644, 0.684 and 0.759 in the test group, and 0.728, 0.764, and 0.805 in the entire group, respectively (Figure 5D). Next, based on clinical features and the risk model, a nomogram predicted the 1-, 2-, 3-year survival rates of EC patients (0.897, 0.455 and 0.234 respectively, Figure 6A). The calibration plot proved the accuracy of the nomogram for the survival prediction (Figure 6B).

**FIGURE 7:**
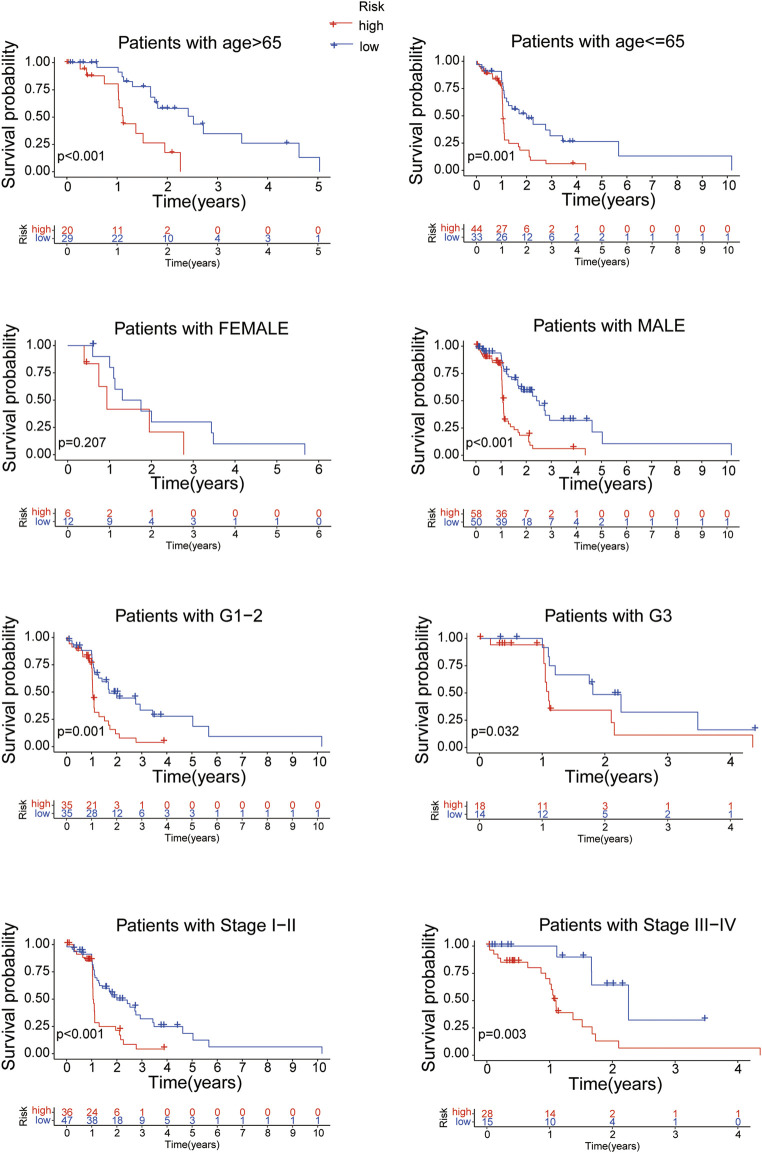
The overall survival of EC patients grouped according to clinical characteristics between the high- and low-risk groups.

Further more, stratifcation analysis investigated the prognostic signifcance of the risk model in subgroups. We analyzed the overall survival of EC patients classified by clinical characteristics (age, gender, tumor grade and tumor stage) between the high- and low-risk groups. It revealed that the high-risk patients presented a shorter overall survival than the low-risk patients (*p* < 0.05) except for the classification of Female probably due to the small number of EC patients (Figure 7). All of the above results confirmed that the 8 proteins risk model could be one independent and superior biomarker for prognosis prediction of EC patients.

### Overall survival analysis of esophageal cancer patients grouped by different expressions of 8 proteins

**FIGURE 8:**
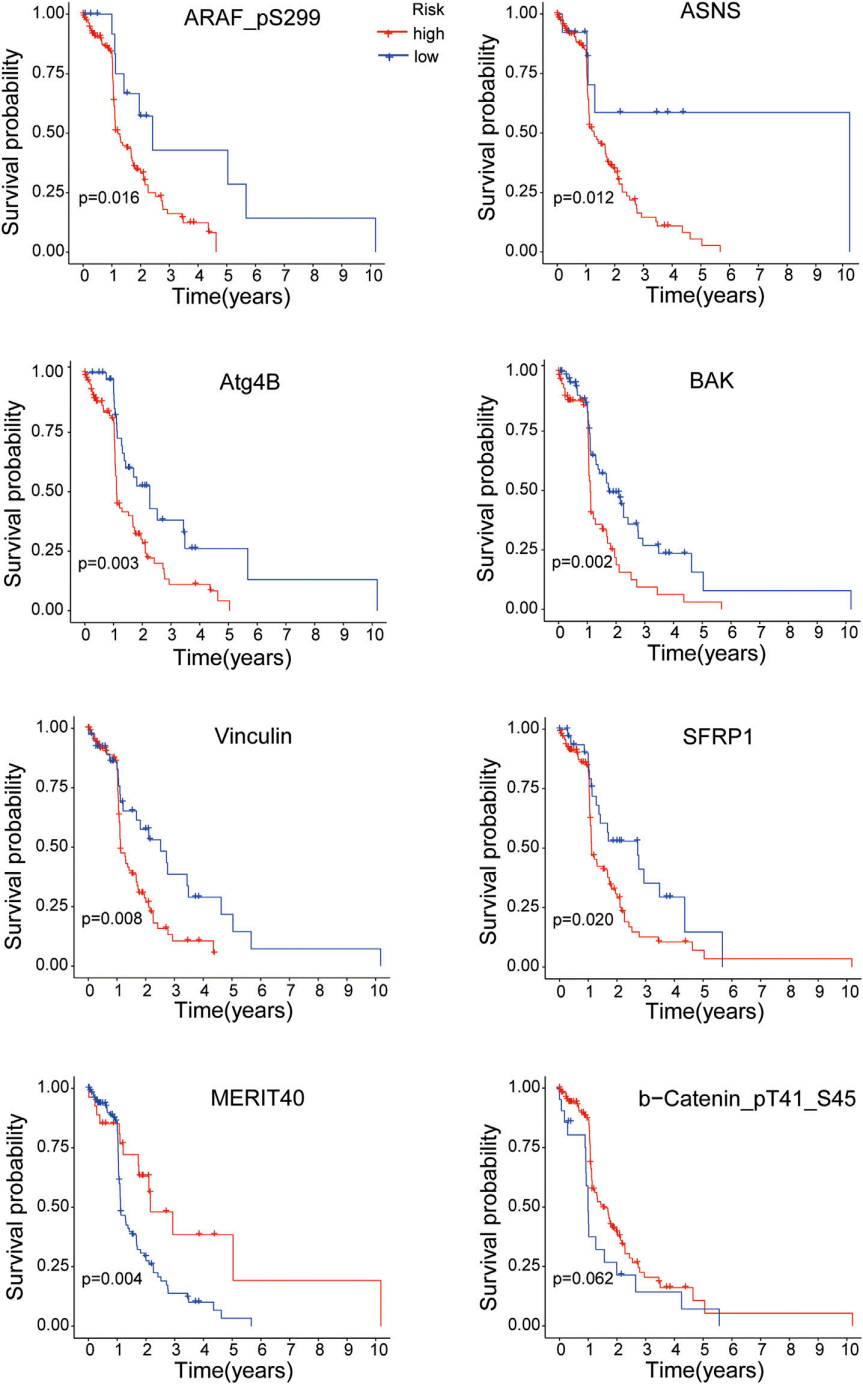
The overall survival analysis of the 8 proteins expressions in EC patients.

K-M survival analysis was performed to research the correlation between the expressions of the 8 proteins and overall survival of EC patients. All samples were divided into the high-expression and low-expression groups according to the medium value of protein expression. As shown in Figure 8, the EC patients with high expressions of ARAF_pS299, ASNS, Atg4B, BAK, Vinculin and SFRP1 proteins had poorer survival time (all *p* < 0.05), while the overall survival of EC patients was better with the high expressions of MERIT40 (*p* < 0.05) and b-Catenin_pT41_S45 (although *p* = 0.062).

### Gene set enrichment analyses analysis and protein interaction network

**FIGURE 9:**
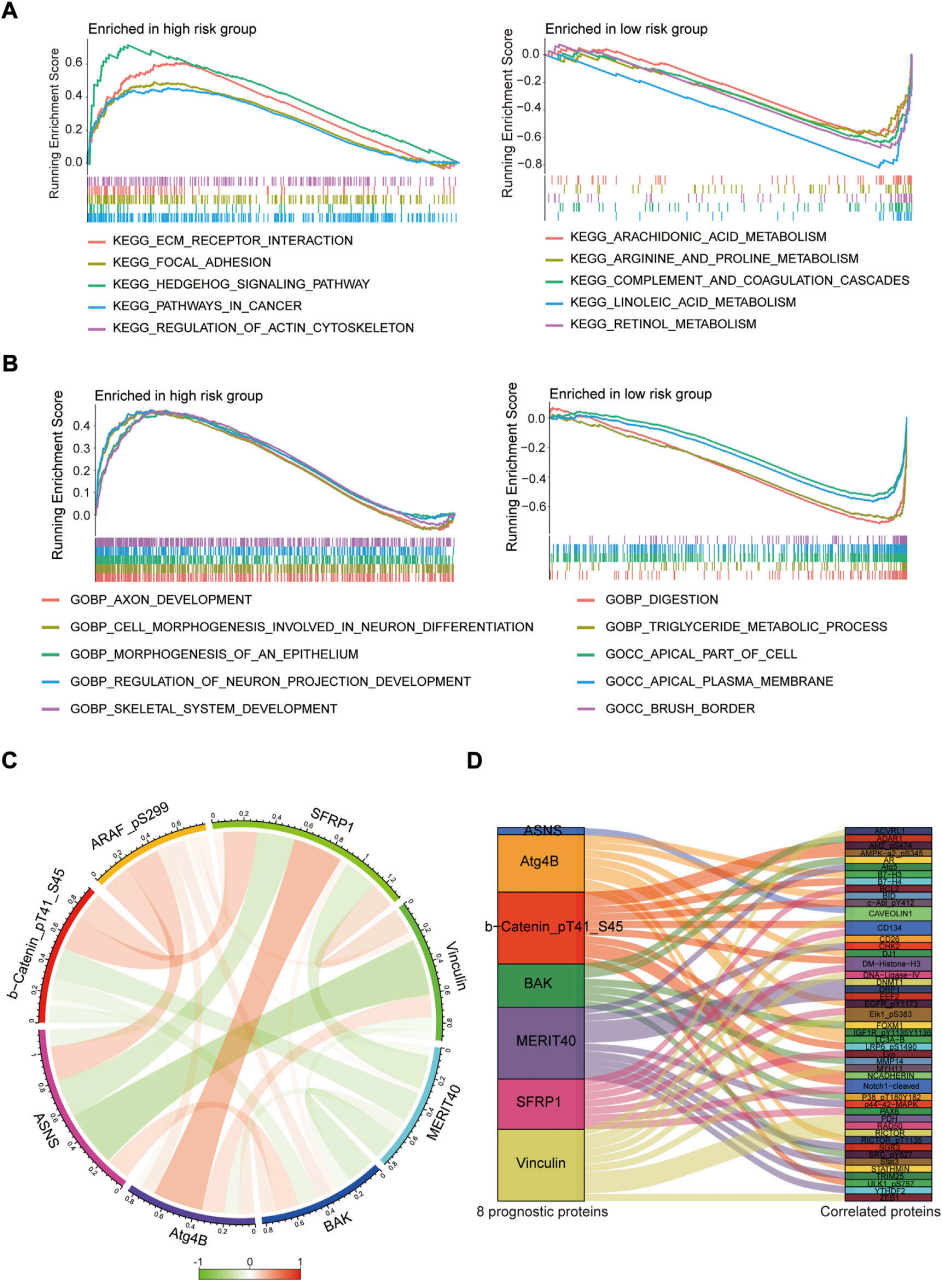
Pathway enrichment analysis and protein interaction network. **(A,B)** The top 5 KEGG and GO pathways enriched in the high- and low-risk groups. **(C)** The interaction relationships among 8 prognostic proteins in circos plot. **(D)** Analysis of co-expressed proteins in EC in sankey diagram.

To investigate the biological functions of the protein prognostic risk model, the significantly enriched KEGG pathways in the risk groups were explored by screening the gene set (c2.cp.kegg.symbols.gmt) in gene set enrichment analyses (GSEA) 4.2.3 software. The top 5 KEGG pathways mainly enriched in the high-risk group were Hedgehog signaling pathway, ECM receptor interaction, Regulation of actin cytoskeleton, Focal adhesion and Pathways in cancer, while Retinol metabolism, Linoleic acid metabolism, Complement and coagulation cascades, Arginine and proline metabolism, Arachidonic acid metabolism were the top 5 KEGG pathways mainly enriched in the low-risk group (all *p* < 0.05, |NES| > 1.5, Figure 9A; Supplementary Table S6). The top 5 GO pathways mainly enriched in the high-risk group were Axon development, Cell morphogenesis involved in neuron differentiation, Morphogenesis of an epithelium, Regulation of neuron projection development and Skeletal system development, while Digestion, Triglyceride metabolic process, Apical part of cell, Apical plasma membrane and Brush border were the top 5 GO pathways mainly enriched in the low-risk group (all *p* < 0.05, |NES| > 1.5, Figure 9B; Supplementary Table S7). The interaction relationships among 8 prognostic proteins in the risk model were shown in Figure 9C. The mutually regulated connection between those prognostic proteins and other proteins expressed in the EC was visualized in Sankey diagram (Figure 9D).

### Tumor immunology and exploration of potential therapeutic agents

**FIGURE 10:**
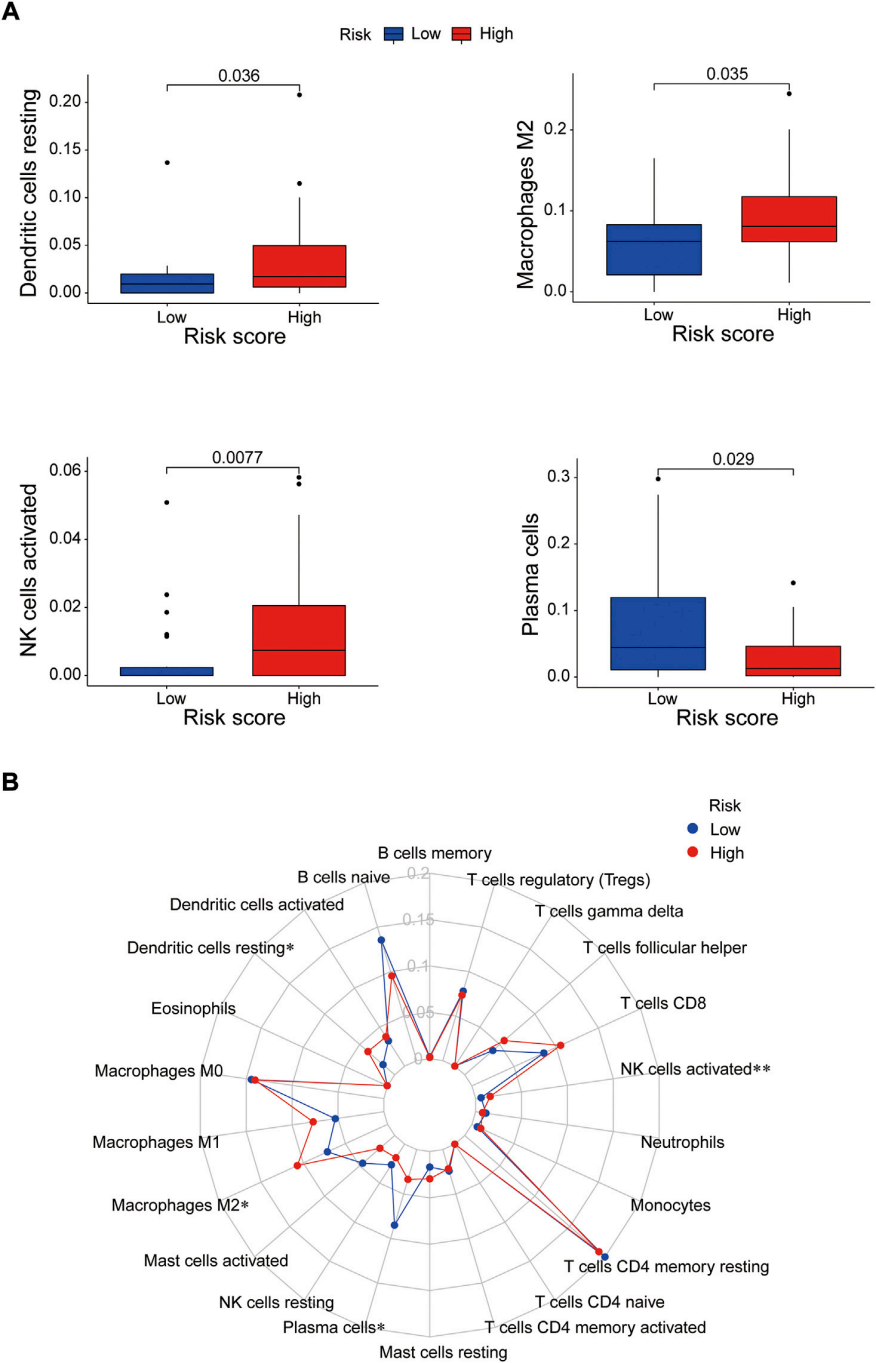
The correlation between immune cells and the risk groups. **(A)** Activated immune cells in the high- and low-risk groups. **(B)** Radar plot. *p < 0.05; **p < 0.01.

**FIGURE 11:**
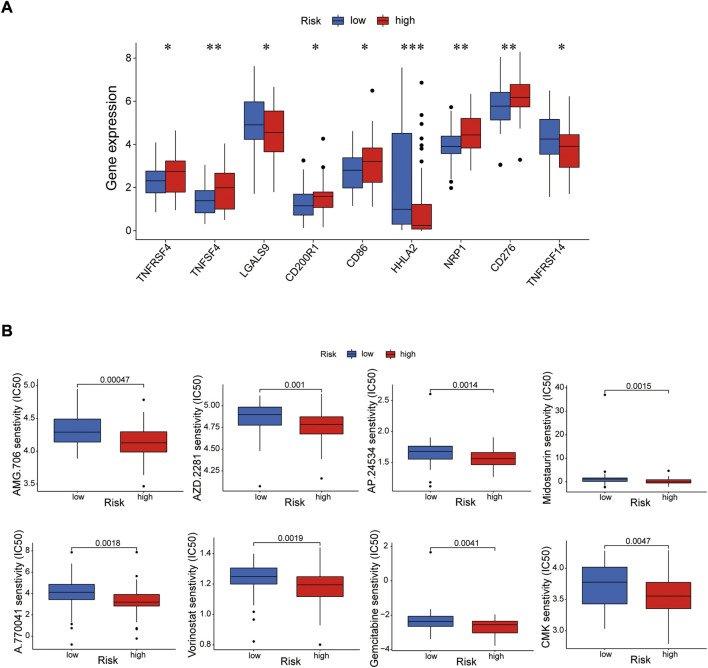
Investigation of tumor immunotherapy. **(A)** The analysis of immune checkpoints in the risk model. **(B)** The prediction of potential therapeutic drugs. *p < 0.05; **p < 0.01; ***p < 0.001.

Immune cells can recognize tumorigenesis factors secreted by tumor cells and infiltrate into the tumor microenvironment to cooperatively regulate tumor growth, immune escape, invasion and metastasis (Sabado et al., 2017; Galluzzi et al., 2020). As shown as Figures 10A,B, through comparing with the low-risk group, the high-risk group presented upregulation of dendritic cells resting, macrophages M2 and NK cells activated, and downregulation of plasma cells *via* GSEA analysis (Supplementary Table S8). Blocking immune checkpoints has become one of many effective strategies to activate antitumor immunity. Our analysis indicated that most of immune checkpoints (TNFRSF4, TNFSF4, CD200R1, CD86, CD276, NRP1) were activated in the high-risk group, while LGALS9, HHLA2 and TNFRSF14 presented high activity in the low-risk group (Figure 11A). It implied that appropriate checkpoint inhibitors could be chosen as a treatment for EC patients depending on the risk model. Further more, the prediction of potential therapeutic drugs showed that AMG.706, AZD.2281, AP.24534, Midostaurin, A.770041, Vorinostat, Gemcitabine and CMK recommended in clinical guidelines were more appropriate treatment for the low-risk patients based on the IC50 values of different risk groups (Figure 11B). These studies could provide an effective and excellent strategy for clinical therapy of EC patients and promoted the development of cancer immunotherapies.

## Discussion

Compared with genomics and transcriptomics, proteomics is more efficient in identifying proteins and pathways that are dysregulated in cells under physiological and pathological conditions, helping to discover disease-specific mutations and epigenetic changes (Duarte and Spencer, 2016). Proteomic analysis of human cells, tissues and blood can provide a valuable strategy for exploring the complex biological processes of human beings, which is helpful for understanding how genetic and non-genetic factors affect the outcome of diseases, so as to reveal biological pathways and biomarkers related to diseases and discover new drug targets for diseases (Suhre et al., 2021). Genomic analysis has broadened our understanding of the molecular pathology in EC (Hanash et al., 2008; Tan et al., 2012; Vogel and Marcotte, 2012; Song et al., 2014; Cancer Genome Atlas Research Network et al., 2017; Suhre et al., 2021). Nevertheless, proteomic analysis provides new insights into the biology of this malignancy. So far, only two studies have used proteomic data to establish prognostic risk models for stomach adenocarcinoma and breast cancer (Huang et al., 2022; Zheng et al., 2022). In this study, construction of a protein risk signature proposes a new method to evaluate its prognostic power for EC.

We obtained 20 proteins differentially expressed between EC and normal tissues, in which 8 proteins (ASNS, b-Catenin_pT41_S45, ARAF_pS299, SFRP1, Vinculin, MERIT40, BAK, Atg4B) were screened out to construct the protein prognostic risk model *via* univariate, multivariate Cox and Lasso regression analyses. Combined with the clinical information, we further comprehensively assess the reliability of the protein prognostic risk model *via* PCA, univariate, multivariate Cox regression analyses, ROC curves and the constructed nomogram, which proved the risk model as an independent and superior biomarker for prognosis prediction of EC patients.

Asparagine synthetases (ASNS) is a catalytic enzyme encoding aspartic acid biosynthesis (Richards and Schuster, 1998), which of the transcription is highly regulated by the cellular trophic state. Early studies have shown that upregulated expression of ASNS could be related to the resistance of leukemia cells to L-aspartase, which is widely used as an active component in the treatment of pediatric acute lymphocytic leukaemia and some types of acute myeloid leukaemia (Zwaan et al., 2002). Similarly, ASNS has been considered as potent biomarker for predicting L-aspartase activity in ovarian cancer cells (Zhang et al., 2013). In addition, it has been shown that ASNS could promote the proliferation and migration abilities of EC cells in the absence of glucose, and clinical data show that EC tissues from the patients with advanced stage or distant metastasis exhibit high level of ASNS expression, which is suggested that ASNS may be a potential target for EC treatment (Fang et al., 2020).

b-Catenin_pT41_S45 (β-catenin/CTNNB1) is one member of the catenin family. CTNNB1, as a key component of E-cadherin/catenin complex, mainly acts as the adhesion effect between cells and cytoplasm. CTNNB1 also plays an important role in Wnt signaling pathway. As the key point of classical Wnt signaling pathway, CTNNB1 gene can bind with transcription factors TCF/LEF, thus forming a complex to promote the up-regulation of target genes, which finally causes a series of biological changes in cells and tissues (Nabais et al., 2003; Wu et al., 2016a). Through activation of Wnt signaling pathway, CTNNB1 involves in the early process of malignant tumors (Spitzner et al., 2021; Yu et al., 2021), including liver cancer, gastric cancer and EC with high incidence, which are all present in ectopic expression of CTNNB1.

ARAF located on human chromosome band Xp11.3 belongs to the serine/threonine protein kinase gene family (Rauch et al., 2010). Similar to other RAF family members, ARAF transduces mitogen-activated protein kinase (MAPK) signaling, thus promoting cell proliferation, differentiation, migration and survival. The RAS-RAF-MEK-ERK cascade has been identified as a therapeutic target in various cancers (Lee and States, 2000; Miura et al., 2014). Early studies on the RAF family focus on B-Raf and C-Raf kinases, resulting in little understanding of the biological function of ARAF. Recent studies have indicated that ARAF required for MAPK activation enhances the migration and invasive ability of various cancer types, such as colon cancer, pancreatic cancer and breast cancers (Lee et al., 2010; Mooz et al., 2014). In addition, ARAF mutation has been discovered in lung cancer, and sorafenib, the RAF-targeted kinase inhibitor, improves the prognosis of advanced lung cancer patients (Imielinski et al., 2014). These findings suggest that ARAF may be a therapeutic target in numerous cancers.

Secreted frizzled-related protein 1 (SFRP1), a member of the SFRP family that contains a cysteine-rich domain, acts as a soluble modulator of Wnt signaling by directly interacting with Wnt (Nakajima et al., 2009), and plays a role in regulating the growth and differentiation of specific tumor cells. Compared with normal tissues, the SFRP1 promoter is found to be highly methylated in EC tissues, resulting in greatly reduced expression level of SFRP1. The detection of circulating methylated SFRP1 in the serum may be a useful biomarker for EC patients (Meng et al., 2011).

Vinculin is an abundant, prominent and well-characterised F-actin binding protein localised in focal adhesions as well as in cell-adherence junctions (Goldmann et al., 2013). The change of Vinculin protein expression has an important effect on the physiological function of the body (Chen et al., 2002; Mierke et al., 2008; Möhl et al., 2009). Vinculin is a major player in cell-matrix adhesion and intercellular adhesion, regulates the migration and invasion of tumor cells (Liu et al., 2007). It is reported that Vinculin presents low expression in non-small cell lung cancer, prostate cancer and colon cancer, and is closely related to prognosis. The Vinculin gene acts as a tumor suppressor gene and affects the occurrence, development, metastasis and invasion of tumors (Gkretsi and Stylianopoulos, 2018).

MERIT40 (BABAM1), as a RAP80-associated protein, is named as an important component of BRISC (Brcc36 isopeptidase complex) and BRCA1 (BReast-CAncer susceptibility gene 1) DNA damage repair complex A. When DNA damage occurs, BABAM1 helps to locate the repair complex to the site of damage, stabilizes the structure of the complex to cause ubiquitination at the site of damage, and trigger cell cycle G2 arrest (Feng et al., 2009; Nikkilä et al., 2009; Shao et al., 2009). Based on the genetic screening of clinical cases and the results of high-throughput gene sequencing, the single nucleotide polymorphisms caused by the nucleotide sequence mutation of BABAM1 on the chromosome had a great correlation with the incidence of breast cancer. It implies BABAM1 plays a role in the occurrence and progression of breast cancer (Caswell et al., 2015).

Bak (Bcl-2 homologous antagonist/killer), is a newly cloned pro-apoptotic member of the Bcl-2 (B-cell lymphoma-2) family. BAK protein binds to Bcl-XL, another member of the Bcl-2 family, through its BH3 domain, which induces the release of Ced-4 homolog proteins and caspase precursors, and Cytochrome C escape from mitochondria, eventually leads to caspase-3 activation and cleavage of specific protein substrates to induce apoptosis (Luna-Vargas and Chipuk, 2016). Studies have found that the Bak gene is expressed in all normal cells, but its expression is out of control in most cancer cells (Luo et al., 2015; Liu et al., 2021). For the mechanism of cancer drug therapy, Bak gene expression is significantly increased in the apoptosis induced by Sulinade, Perillyl alcohol, Butyric acid, interferon and many other drugs (Anis et al., 2020; Li et al., 2020). Therefore, BAK may become an important effector gene in tumor gene therapy.

Atg4B, as a member of Atg4 family, exerts a vital role in autophagosome production and maturation. Compared with other family members, Atg4B has stronger enzyme activity and a wider range of substrate recognition, which is the most important enzyme in regulating autophagy (Li et al., 2011). Numerous studies have shown that regulation of Atg4B expression can affect the occurrence and development of cancer. For example, ATG4B is highly expressed in lung cancer tissues (Wu et al., 2016b); targeting Atg4B inhibits autophagy and reduces tumor cell survival in chronic myeloid leukemia (Rothe et al., 2014); overexpression of the dominant negative mutant Atg4B^C74A^ in prostate cancer inhibits cell proliferation and enhances chemotherapeutic drug sensitivity (Tran et al., 2013).

Based on previous studies, all of the prognosis-related proteins play a role in various tumors to a certain extent. Our study reveals the prognostic role of these proteins as a risk model in EC. To further explore the effective potential therapies of EC, we found three types of immune cells (Dendritic cells resting, macrophages M2 and NK cells activated) were highly expressed in the high-risk group, and only one type of immune cell (plasma cells) was highly expressed in the low-risk group. Our data also showed that multiple immune checkpoints, such as TNFRSF4, TNFSF4, CD200R1, CD86, CD276, NRP1, presented strong activity in the high-risk group, while TNFSF4, HHLA2 and TNFRSF14 were activated in the low-risk group. It implied that we could choose appropriate immune cell therapy drugs and checkpoint inhibitors as a treatment for EC patients depending on the risk mode. Further more, the prediction of potential therapeutic drugs showed that AMG.706, AZD.2281, AP.24534, Midostaurin, A.770041, Vorinostat, Gemcitabine and CMK recommended in clinical guidelines were more appropriate treatment for the low-risk patients.

In summary, we used the proteome profiling and clinical information of EC patients in TCGA database to construct and assess a novel 8 proteins risk model for prognosis prediction in EC. Through various analysis and verification, this prognostic model could act as a good prognostic evaluation for EC patients and provide new insight into the diagnosis and treatment of EC.

## Data Availability

The original contributions presented in the study are included in the article/Supplementary Material; further inquiries can be directed to the corresponding authors.

## Supplementary material

Supplementary Table S1 The proteome profiling containing 126 EC cases.

Supplementary Table S2 The clinical information containing 185 EC patients from the TCGA database.

Supplementary Table S3 The list of 126 intersecting samples after matching the proteome data and clinical data.

Supplementary Table S4 The list of 20 proteins significantly associated with survival time of EC patients by univariate Cox regression analysis.

Supplementary Table S5 The list of 14 proteins related to survival state of EC patients by the Lasso regression analysis.

Supplementary Table S6 The list of KEGG pathways in the risk model.

Supplementary Table S7 The list of GO pathways in the risk model.

Supplementary Table S8 The list of immune cells activated in the high- and low-risk groups.
